# The Birth Weight Lowering C-Allele of rs900400 Near *LEKR1* and *CCNL1* Associates with Elevated Insulin Release following an Oral Glucose Challenge

**DOI:** 10.1371/journal.pone.0027096

**Published:** 2011-11-04

**Authors:** Ehm A. Andersson, Marie N. Harder, Kasper Pilgaard, Charlotta Pisinger, Alena Stančáková, Johanna Kuusisto, Niels Grarup, Kristine Færch, Pernille Poulsen, Daniel R. Witte, Torben Jørgensen, Allan Vaag, Markku Laakso, Oluf Pedersen, Torben Hansen

**Affiliations:** 1 Novo Nordisk Foundation Center for Basic Metabolic Research, Faculty of Health Sciences, University of Copenhagen, Copenhagen, Denmark; 2 Steno Diabetes Center, Gentofte, Denmark; 3 Research Centre for Prevention and Health, Glostrup University Hospital, Glostrup, Denmark; 4 Department of Medicine, University of Eastern Finland and Kuopio University Hospital, Kuopio, Finland; 5 Department of Public Health, Faculty of Health Science, University of Copenhagen, Copenhagen, Denmark; 6 Department of Endocrinology, Rigshospitalet, Copenhagen, Denmark; 7 Institute of Biomedical Science, Faculty of Health Sciences, University of Copenhagen, Copenhagen, Denmark; 8 Faculty of Health Sciences, University of Aarhus, Aarhus, Denmark; 9 Hagedorn Research Institute, Gentofte, Denmark; 10 Faculty of Health Sciences, University of Southern Denmark, Odense, Denmark; Peninsula College of Medicine and Dentistry, -University of Exeter, United Kingdom

## Abstract

**Background and Aim:**

The first genome-wide association study on birth weight was recently published and the most significant associated birth weight lowering variant was the rs900400 C-allele located near *LEKR1* and *CCNL1*. We aimed to replicate the association with birth weight in the Danish Inter99 study and furthermore to evaluate associations between rs900400 and indices of insulin secretion and insulin sensitivity obtained by oral glucose tolerance tests in adults from the Danish Inter99 study and the Finnish, Metabolic Syndrome in Men (METSIM) sample.

**Methods:**

For 4,744 of 6,784 Inter99 participants, midwife journals were traced through the Danish State Archives and association of rs900400 with birth weight was examined. Associations between rs900400 and fasting serum insulin, fasting plasma glucose, insulinogenic index, homeostasis model assessment of insulin resistance (HOMA-IR) and disposition index were studied in 5,484 Danish and 6,915 Finnish non-diabetic individuals and combined in meta-analyses.

**Results:**

The C-allele of rs900400 was associated with a 22.1 g lower birth weight ([−41.3;−3.0], *P* = 0.024) per allele. Moreover, in combined analyses of the Danish Inter99 study and the Finnish METSIM study we found that the birth weight lowering allele was associated with increased insulin release measured by the insulinogenic index (β = 2.25% [0.59; 3.91], *P* = 0.008) and with an increased disposition index (β = 1.76% [0.04; 3.49], *P* = 0.05).

**Conclusion:**

The birth weight lowering effect of the C-allele of rs900400 located near *LEKR1* and *CCNL1* was replicated in the Danish population. Furthermore the C-allele was associated with increased insulin response following oral glucose stimulation in a meta-analysis based on Danish and Finnish non-diabetic individuals.

## Introduction

Birth weight is a crude measure of the entire period of pre-natal growth. It is a complex trait influenced by multiple intrauterine factors as well as the genetic disposition of both the fetus and the mother. It is well known that low birth weight is associated with adult-onset metabolic diseases including type 2 diabetes (T2D) [1], [2] and it has been hypothesized that this link is due to foetal growth restriction being detrimental for the natural organ development [3]. Besides, recent data provided proof-of-concept for the idea that the relationship between low birth weight and risk of T2D to some extent may be explained by common genetic disposition to both traits (*the fetal insulin hypothesis*) [4]. This hypothesis is based on insulin being an important fetal growth factor, and thus genetic variation impairing insulin secretion or action may result in both reduced fetal growth and risk of T2D. Thus, lower birth weight was found among carriers of T2D risk alleles in or near *HHEX-IDE*, *CDKAL1* and *ADCY5* [5], [6], [7], [8], [9], however only the latter was confirmed at genome-wide significance. Risk alleles in *HHEX-IDE* and *CDKAL1* confer increased risk of T2D due to lower insulin secretion [10], [11] and the birth weight lowering effect of these two alleles suggests that insulin secretion is diminished already in pre-natal life. This may as well be the case for the *ADCY5* risk allele, but the exact mechanism by which this variant predisposes to both low birth weight and T2D is yet to be determined [12]. However, several T2D risk alleles affecting adult insulin secretion have not been found to have an effect on birth weight. Variants in the *FTO* and *TCF7L2* loci have been tested in large cohorts [13], [14], but it cannot be excluded that for other variants the lack of association with birth weight might be due to missing power.

The first genome-wide association (GWA) study on birth weight demonstrated that rs9883204 located in *ADCY5* and rs900400 located near *LEKR1* and *CCNL1* were associated with a decrease in birth weight by 0.063 and 0.086 z-score units per allele, respectively [5].

By investigating published GWA-databases from the GIANT and MAGIC consortia the authors found that, while the *ADCY5* locus was related to T2D, rs900400 was not associated with either T2D, height, BMI, or fasting glyceamic traits [5]. So far, genetic variants in or near *LEKR1* and *CCNL1* have not been reported to be associated with adult metabolic phenotypes. Due to limited knowledge about this novel signal, we aimed to confirm the association between rs900400 and birth weight in the Danish population and furthermore to evaluate associations between rs900400 and five indices of insulin release and insulin sensitivity obtained from an oral glucose tolerance test in adults of the Danish Inter99 population and the Finnish Metabolic Syndrome in Men (METSIM) study as well as in combined analyses.

## Materials and Methods

### Study population

#### Inter99

Ethics statement: All participants gave written informed consent and the protocol was in accordance with the Helsinki Declaration, and approved by Copenhagen County ethic committee (ClinicalTrials.gov NCT00289237).

Individuals examined in the present study were from the Danish Inter99 study, which at baseline comprised 6,784 individuals living in the region of Copenhagen. The Inter99 study is a population-based, randomised non-pharmacological intervention study of prevention of ischemic heart disease conducted at the Research Centre for Prevention and Health in Glostrup, Denmark (www.inter99.dk) [15], [16]. For 4,744 participants, midwife journals were traced through the Danish State Archives. These journals contained information on mother's age, parity and marital status as well as birth weight, length at birth and prematurity of the newborn [17]. Ponderal index was calculated as birth weight (kg) / birth length (m)^3^. Birth weight characteristics of participants included in the study can be seen in Table 1. Information about maternal diabetes status (yes/no/unknown) was obtained by a questionnaire during the baseline visit in 1999–2001. The age of onset of maternal diabetes was not registered. Term birth was defined by a gestational week between 37 and 41. Preterm singleton deliveries (n = 446) and individuals born from multiple pregnancies (n = 85) were excluded. The final number of individuals included in the analyses of birth weight was 4,210.

**Table 1:** Birth weight characteristics of participants from the Inter99 study and characteristics of non-diabetic participants from the Inter99 study and from the METSIM study.

Inter99	N	Inter99	N	METSIM
Years of birth		1939–1970		
Birth weight (g)	4210	3488±450		
Birth lenght (cm)	4197	52±2		
Ponderal index	4197	24.8±2.3		
Age (years)	5484	46.0±7.9	6915	57.1±7.0
Body mass index (kg/m^2^)	5483	26.0±4.4	6912	26.8±3.8
Fasting insulin (pmol/l)	5292	40.6±26.1	6911	49.1±34.0
Fasting plasma glucose (mmol/l)	5479	5.5±0.5	6915	5.7±0.5
HOMA-IR (mmol/l*pmol/l)	5290	1.7±1.1	6911	2.1±1.5
Insulinogenic index (pmol/l / mmol/l)	5040	29.8±19.6	6876	39.9±30.1
Disposition index	5040	22.6±16.8	6876	22.6±16.0

Data are mean ± standard deviation or median (interquartile range).

Association between the rs900400 genotype and quantitative diabetes-related traits were studied in 5,484 non-diabetic individuals from the population-based Inter99 sample of Danish individuals aged 30–60 years. All participants had an oral glucose tolerance test (OGTT). Included in the analysis were 4,329 individuals with normal glucose tolerance (NGT), 489 individuals with impaired fasting glycaemia (IFG) and 666 individuals with impaired glucose tolerance (IGT). Patients in the Inter99 sample with diabetes were not included in the present analysis of quantitative traits. All individuals were Danes by self report.

#### METSIM

Ethics statement: Written informed consent has been obtained from each participant after the purpose and the risks/benefits of the study have been explained. The study was approved by the Ethics Committee of the University of Kuopio and Kuopio University Hospital, and it was in accordance with the Helsinki Declaration.

Individuals from the population-based cross-sectional Metabolic Syndrome in Men (METSIM) study comprise 10,197 men aged 45 to 70 years. The men have been randomly selected from the population register of the town of Kuopio in eastern Finland (population 95,000). Every participant had a 1-day outpatient visit to the Clinical Research Unit at the University of Kuopio, including an interview on the history of previous diseases and current drug treatment and an evaluation of glucose tolerance and cardiovascular risk factors. No birth weight data were available for the participants. Associations between the rs900400 genotype and quantitative diabetes related traits were studied in 6,915 non-diabetic individuals. Of these participants, 4,638 had normal glucose tolerance, 1,206 isolated impaired fasting glucose (IFG), 630 isolated impaired glucose tolerance (IGT), 441 a combination of IFG and IGT according to WHO 1997 criteria [18].

### Biochemical measures

#### Inter99

Blood samples were drawn after an overnight fast followed by an OGTT. Plasma glucose was analyzed by a glucose oxidase method (Granutest; Merck, Darmstadt, Germany). Serum insulin ((excluding des-31,32) and intact proinsulin) was measured using the AutoDELFIA insulin kit (Perkin-Elmer, Wallac, Turku, Finland).

#### METSIM

Blood samples were drawn after 12 h of fasting followed by an OGTT. Plasma glucose was measured from plasma (FC-mixture: NaF/ Na-Citrate/ EDTA-Na2) by Enzymatic photometric test, Glucose hexokinase. Reagent: Konelab System Reagents, Glucose (HK), Thermo Fisher Scientific, Vantaa, Finland. Instrumentation: Konelab 20XTi Clinical Chemistry Analyzer, Thermo Fisher Scientific, Vantaa, Finland. Insulin was measured in mU/l units (converted to pmol/l multiplying by 6) from plasma (EDTA) by Immunoassay, luminometric measurement. Reagent: ADVIA Centaur Insulin IRI, no 02230141, Siemens Medical Solutions Diagnostics, Tarrytown, NY, USA. Instrumentation: Siemens ADVIA Centaur®, Siemens Medical Solutions Diagnostics, Tarrytown, NY, USA.

#### Indices of insulin release and insulin sensitivity for both study samples

Oral glucose-stimulated insulin release was reported as the insulinogenic index. The insulinogenic index was calculated as (serum insulin at 30 minutes [pmol/l]-fasting serum insulin [pmol/l]) / plasma glucose at 30 minutes (mmol/l). Homeostasis model assessment of insulin resistance (HOMA-IR) was calculated as ((fasting plasma glucose (mmol/l) * fasting serum insulin (pmol/l)) / 135) for the Inter99 cohort and as ((fasting plasma glucose (mmol/l) * fasting plasma insulin (µU/l))/ 22.5) in the METSIM cohort. In order to construct an OGTT-based disposition index, we divided insulinogenic index with the reciprocal of HOMA-IR (Insulinogenic index/HOMA-IR). Clinical characteristics of the Inter99 and METSIM individuals are shown in Table 1.

### Genotyping

#### Inter99

Genotyping was performed using KASPar® genotyping (KBioscience, Hoddesdon) with a success rate of 98.3% and an error rate of 0% in 454 duplicate samples. There was a slight deviation from Hardy Weinberg equilibrium (*P* = 0.011).

#### METSIM

Genotyping was performed using the Applied Biosystems TaqMan Allelic Discrimination Assay with a success rate of 98.1% and an error rate of 0% in 4.5% duplicate samples. The genotype distribution was consistent with Hardy Weinberg equilibrium (*P* = 1.0).

### Statistical analysis

#### Inter99

All statistical analyses were performed using RGui version 2.8.1 (available at http://www.r-project.org). The associations of rs900400 with birth weight, length at birth and ponderal index in the Inter99 population were calculated using linear regression models adjusted for sex, maternal diabetes (yes vs. no/NA) and parity (0, 1, 2, 3 or ≥4). Z-scores were calculated as [value-mean]/standard deviation (based on the mean and SD birth weight in the included Inter99 sample).

#### METSIM

Statistical analyses were performed using SPSS version 17 (SPSS, Chicago, IL). Associations with quantitative variables during an OGTT were performed as described below.

#### Both studies

Associations of rs900400 with indices of insulin release and insulin sensitivity were performed by linear regression models adjusted for age or for age and BMI (the analyses were also adjusted for sex in the Inter99 sample). Effect sizes are given as actual values or percentage (%) if the trait was natural logarithmically (ln) transformed. Only additive genetic models were considered assuming a constant change per risk allele and a *P*-value <0.05 was considered significant.

Fixed effect meta-analyses (up to n = 12,394) were performed using effect size estimates and standard errors (SE) derived from linear regression analyses from the Inter99 (adjusted for age and sex) and the METSIM (adjusted for age) populations. The weight of the two studies in the meta-analyses was estimated using inverse variance assuming fixed effects. Heterogeneity was measured by Q-statistics.

Statistical power was estimated using 1000 simulations. We used the empirical variance of the observed traits in the Danish Inter99 cohort to simulate phenotypes from a normal distribution, so that variance across genotypes is drawn from the estimated variance.

In the combined analyses, we have estimated the effect sizes that we have more than 80% statistical power to detect (P<0.05) assuming a minor allele frequency of 40% resembling the HapMap-CEU frequency of rs900400 (Table S1).

## Results

In 4,210 individuals from the Danish Inter99 population the C-allele of rs900400 near *LEKR1* and *CCNL1* was associated with 22.1 g lower birth weight per allele (95% CI −41.3;−3.0, *P* = 0.024). A trend towards a lower ponderal index was also observed (β = −0.08 [−0.18;0.01], *P* = 0.094) for carriers of the C-allele, whereas length at birth was not associated with rs900400 (*P* = 0.230) (Table 2).

**Table 2:** Associations between rs900400 near *LEKR1* and *CCNL1* and birth weight, birth length and ponderal index in the Danish Inter99 sample.

	TT	TC	CC	Effect	Effect z-score	*P*
N	1332	1875	722			
(Men/Women)	(640/692)	(893/982)	(326/396)			
Birth weight (g)	3493±451	3503±446	3435±439	−22.1[−41.3;−3.0]	−0.05[−0.09;−0.01]	0.024
Birth length (cm)	52±2	52±2	52±2	−0.05[−0.13;0.03]	−0.03[−0.07; 0.02]	0.23
Ponderal index (kg/m^3^)	24.8±2.3	24.8±2.3	24.6±2.2	−0.08[−0.18;0.01]	−0.04[−0.08;0.01]	0.094

Effects and P-values are adjusted for sex, maternal diabetes and parity assuming an additive model of inheritance. Ponderal index was calculated as birth weight (kg)/birth length (m)^3^.

**Figure 1:**
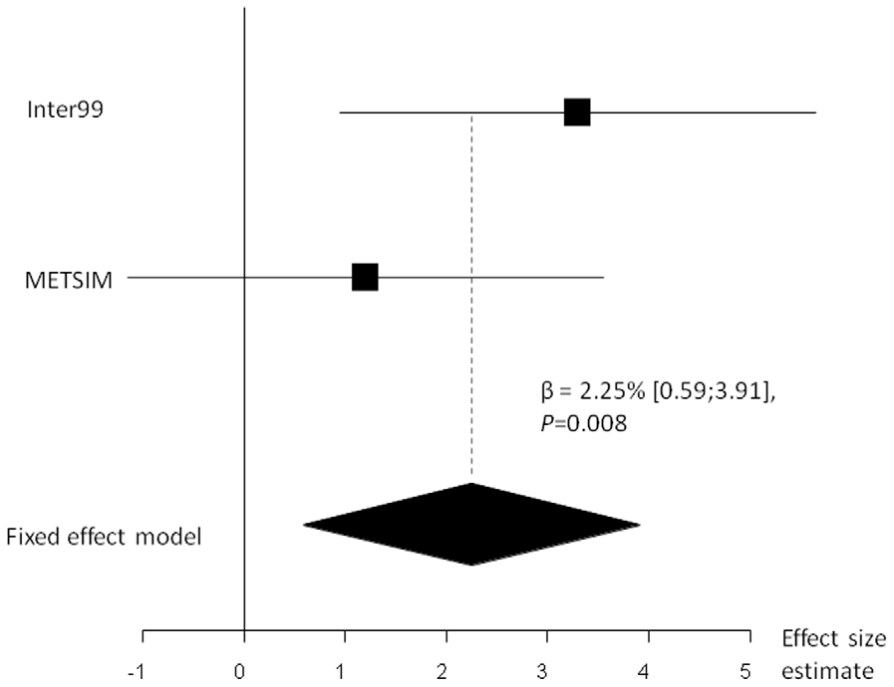
Meta-analysis of rs900400 and insulinogenic index including 11,916 individuals from the Inter99 and METSIM study. Effect size estimates and standard errors are combined in a meta-analysis using the inverse variance method. The black diamond represents the combined change in insulinogenic index per C-allele. Effect size estimate (β) and *P*-value are presented for the combined analysis with 95% confidence interval in square brackets.

In the Inter99 study, we found that the birth weight lowering C-allele was associated with increased insulin release as estimated by the insulinogenic index (β = 3.3% [1.0;5.6], *P* = 0.005) as well as increased disposition index (β = 3.6% [1.0;6.2], *P* = 0.007) (Table 3). Moreover, we found a lower fasting plasma glucose level (−0.02 mmol/l [−0.04;−0.002], *P* = 0.021) for carriers of the birth weight lowering allele in the Inter99 cohort. These observations, except the findings for fasting plasma glucose, were also observed in the subgroup of individuals with normal glucose tolerance in Inter99 (data not shown). The C-allele of rs900400 was not significantly associated with any of the five traits in the METSIM population (table 3). However, the directions of the associations with insulinogenic index and disposition index were similar to what was observed in the Inter99 sample although the effect size estimates were considerably smaller in the METSIM population. Additional adjustment for BMI in all analyses did not change the level of significance. Fixed effect meta-analyses of data from the Inter99 and METSIM studies were performed for all five metabolic traits (fasting serum insulin, fasting plasma glucose, insulinogenic index, HOMA-IR and disposition index) including up to 12,394 individuals. In combined meta-analyses the C-allele of rs900400 was associated with increased insulin response to an oral glucose load estimated by the insulinogenic index (β = 2.25% [0.59; 3.91], *P* = 0.008, figure 1 and table 4) and with an increased disposition index in C-allele carriers (β = 1.76% [0.04; 3.49], *P* = 0.05, table 4). No other traits were associated with rs900400 in the meta-analyses (table 4). No heterogeneity was observed in any of the meta-analyses (*P*>0.05).

**Table 3:** Associations between rs900400 near *LEKR1* and *CCNL1* and quantitative metabolic traits during an oral glucose tolerance test in 5,484 non-diabetic participants of the Inter99 study and 6,915 non-diabetic men from the METSIM study.

Inter99	TT	TC	CC	Effect [95% CI]	P
N (Men/Women)	1908(926/982)	2584(1297/1287)	992(463/529)		
Age (years)	46±8	46±8	45±8		
Fasting serum insulin (pmol/l)	40.91±26.05	40.09±26.16	41.05±26.09	−0.3% [−2.4;1.9]	0.81
Fasting plasma glucose (mmol/l)	5.47±0.51	5.46±0.51	5.41±0.52	−0.02 mmol/l [−0.04;0.0]	0.021
HOMA-IR (µU/l * mmol/l)	1.68±1.13	1.65±1.14	1.67±1.12	−0.6% [−2.9;1.7]	0.62
Insulinogenic index (pmol/l / mmol/l)	29.07±18.26	29.51±19.36	31.69±22.14	3.3% [1.0;5.6]	0.005
Disposition index (Insulinogenic index / HOMA-IR)	21.91±15.92	22.5±16.32	23.89±19.23	3.6% [1.0;6.2]	0.007

METSIM	TT	TC	CC	Effect [95% CI]	P
N	3331	2934	647		
Age (years)	57.0±6.9	57.1±7.0	57.5±7.0		
Fasting plasma insulin (pmol/l)	48.88±33.51	49.19±34.44	50.04±34.61	0.9% [−1.2;3.0]	0.419
Fasting plasma glucose (mmol/l)	5.70±0.48	5.71±0.49	5.70±0.47	0.00 mmol/l [−0.02;0.02]	0.982
HOMA-IR (µU/l *mmol/l)	2.10±1.53	2.11±1.55	2.14±1.52	0.9% [−1.4;3.1]	0.445
Insulinogenic index (pmol/l / mmol/l)	39.55±29.53	40.03±30.53	40.92±30.93	1.2% [−1.2;3.5]	0.340
Disposition index (Insulinogenic index / HOMA-IR)	22.56±16.25	22.70±15.81	22.32±15.34	0.2% [−2.1;2.4]	0.890

Data are mean±SD and are stratified according to genotype of rs900400. Effects are given as actual values (plasma glucose) or percentage (%) (all other traits) since these traits are logarithmically transformed. Effects and p-values are adjusted for age (and sex in Inter99) assuming an additive model of inheritance.

**Table 4:** Meta-analyses of rs900400 and insulin traits including up to 12,394 individuals from the Inter99 and the METSIM study.

Combined meta-analyses	N	Effect [95% CI]	P	P (heterogeneity)
Fasting insulin (pmol/l)	12,203	0.3% [−1.22; 1.82]	0.70	0.44
Fasting plasma glucose (mmol/l)	12,394	−0.0088 mmol/l [−0.02; 0.004]	0.19	0.13
HOMA-IR (µU/l * mmol/l)	12,201	0.20% [−1.37; 1.77]	0.81	0.35
Insulinogenic index (pmol/l / mmol/l)	11,916	2.25% [0.59; 3.91]	0.008	0.22
Disposition index (Insulinogenic index / HOMA-IR)	11,916	1.76% [0.04; 3.49]	0.045	0.055

The effect size estimates represents the combined change of the different traits per C-allele. *P*-values are presented for the combined analysis. *P*-values for heterogeneity are also presented.

## Discussion

We confirm the association of rs900400 C-allele near *LEKR1* and *CCNL1* with lower birth weight in the Danish Inter99 study sample. The same allele was found by Freathy *et al.* to be associated with a lower ponderal index in 21,515 European individuals [5]. We also observe a trend towards a lower ponderal index, although this trait does not reach statistical significance in our cohort.

Our confidence interval is just overlapping with the estimated effect size reported by Freathy *et al*, indicating that statistically the effect sizes are similar in the two cohorts. However, the effect size estimates of birth weight and ponderal index in the present study are slightly lower than the combined effects reported by Freathy *et al* [5]. This could be explained by general population differences and slight deviations in linkage equilibrium among the populations, assuming that this polymorphism is not the causative variant. Moreover, we were not able to adjust for gestational age also impeding the accuracy of birth weight as a measure of growth conditions. However, in the GWA meta-analysis three studies without information about gestational age did not introduce heterogeneity in the overall analyses suggesting that this variant is not strongly associated with gestational age [5].

Surprisingly, in meta-analyses based on the Danish Inter99 and Finnish METSIM studies we found an increased oral glucose stimulated insulin release measured by the insulinogenic index and the disposition index in carriers of the birth weight lowering C-allele. The disposition index could be considered as an estimate of the ability of the pancreatic beta cells to respond appropriately to the level of insulin sensitivity. If these data are upheld in larger study samples, it can be suggested that the increased insulin release represents a primary metabolic feature in these individuals. This finding may appear in contrast to what may have been expected according to the fetal insulin hypothesis [4] and based on recent observations for birth weight lowering variants in or near *CDKAL1*, *HHEX-IDE* and *ADCY5* [5]–[9]. Nevertheless, the variant near *LEKR1* and *CCNL1* was not associated with impaired β-cell function or insulin action demonstrating that genetic variants conferring low birth weight may not necessarily be related to an adverse glycemic phenotype. Adult determinants of insulin secretion and insulin action may however not reflect insulin regulation in fetal life. In this study the adult insulin secretion was measured as a response to an oral glucose challenge, which triggers insulin secretion through the gut and not through the umbilical cord like in the uterus. It could also be speculated that the increased secretion may somehow be related to compensatory post-natal mechanisms. Furthermore, it has been seen before that a mutation can cause opposite effects on insulin secretion in early versus later life, since a *HNF4A* mutation causes hyperinsulimea in utero and then later causes diabetes in adulthood [19]. Obviously further genetic epidemiological and metabolic studies in representative study samples are needed to verify the findings. In order to achieve genome-wide significance with 80% statistical power, sample sizes of >56,000 individuals are needed.

In our study, associations to five metabolic traits have been examined. Although some traits are correlated, we recognize the possibility of the associations being false positive findings due to lack of correction for multiple testing. However, if a stringent Bonferroni correction for five independent tests were performed, the association with the insulinogenic index observed in the combined analysis still remains statistically significant.

In conclusion, we confirm the association with lower birth weight for the C-allele of rs900400 located near *LEKR1* and *CCNL1* and we demonstrate a novel association with increased insulin response for carriers of the birth weight lowering allele. The validity of the present findings needs to be tested in future studies.

## Supporting Information

Table S1 Effect sizes that we have 80% statistical power to detect in the combined analyses with a minor allele frequency of 40% and with a *P*-value of 0.05 for the five listed traits.
(DOC)
