# Supplementary material for: The Birth Weight Lowering C-Allele of rs900400 Near LEKR1 and CCNL1 Associates with Elevated Insulin Release following an Oral Glucose Challenge
Source: PLoS One. 2011 Nov 4;6(11):e27096. doi: 10.1371/journal.pone.0027096 (PMC3208566; doi:10.1371/journal.pone.0027096)
Supplement: Table S1 — Effect sizes that we have 80% statistical power to detect in the combined analyses with a minor allele frequency of 40% and with a P-value of 0.05 for the five listed traits. (DOC) [file pone.0027096.s001.doc]

Table S1: Effect sizes that we have 80% statistical power to detect in the combined analyses with a minor allele frequency of 40% and with a *P*-value of 0.05 for the five listed traits.

| **Traits** | **N** | **Effect** |
| --- | --- | --- |
| Fasting insulin (pmol/l) | 12,203 | 2.2% |
| Fasting plasma glucose (mmol/l) | 12,394 | 0.019 mmol/l |
| HOMA-IR (µU/l * mmol/l) | 12,201 | 2.3% |
| Insulinogenic index (pmol/l / mmol/l) | 11,916 | 2.3% |
| Disposition index (Insulinogenic index /HOMA-IR) | 11,916 | 2.6% |
